# Correlation of Load-Bearing Behavior of Reinforced Concrete Members and Velocity Changes of Coda Waves

**DOI:** 10.3390/ma15030738

**Published:** 2022-01-19

**Authors:** Felix Clauß, Niklas Epple, Mark Alexander Ahrens, Ernst Niederleithinger, Peter Mark

**Affiliations:** 1 Institute of Concrete Structures, Faculty of Civil and Environmental Engineering, Ruhr University Bochum, Universitätstraße 150, 44801 Bochum, Germany; Alexander.Ahrens@rub.de (M.A.A.); Peter.Mark@rub.de (P.M.); 2 Federal Institute for Materials Research and Testing (BAM), Unter den Eichen 87, 12205 Berlin, Germany; Niklas.Epple@bam.de (N.E.); Ernst.Niederleithinger@bam.de (E.N.)

**Keywords:** ultrasound, coda wave interferometry, structural health monitoring, strain, stress, reinforced concrete, moment-curvature relationship, tests, cracking, fiber optic sensors

## Abstract

The integral collection of information such as strains, cracks, or temperatures by ultrasound offers the best prerequisites to monitor structures during their lifetime. In this paper, a novel approach is proposed which uses the collected information in the coda of ultrasonic signals to infer the condition of a structure. This approach is derived from component tests on a reinforced concrete beam subjected to four-point bending in the lab at Ruhr University Bochum. In addition to ultrasonic measurements, strain of the reinforcement is measured with fiber optic sensors. Approached by the methods of moment-curvature relations, the steel strains serve as a reference for velocity changes of the coda waves. In particular, a correlation between the relative velocity change and the average steel strain in the reinforcement is derived that covers 90% of the total bearing capacity. The purely empirical model yields a linear function with a high level of accuracy ($R^{2}=0.99,$ RMSE≈90μstrain).

## 1. Introduction

Civil engineers have long been aware of the gradual aging of infrastructure. Global availability of raw materials, ease of workability, almost arbitrary formability, and economic considerations have led to the vast majority of existing structures being made of reinforced concrete (RC). Its increasingly critical state of preservation has driven their activities. Today, regular inspection on-site, i.e., of bridges, is part of the daily business of structural inspectors. However, annual intervals of visual inspection [1] do not reach the root of all problems. Rather, they enable reacting to existing (visible) damage. Until damage becomes visible, time passes by and is lost to take countermeasures early. What can be done in the meantime when damage is not yet visible from the outside? This is where structural health monitoring comes into play. Research streams in in this field are copious (e.g., [2,3,4,5]). Available options to technically equip structures are plentiful (fiber optic sensors (FOS), acoustic emission, strain gauges, displacement transducers, etc.). They address all typical issues of bridges [6] and promise to detect and monitor any changes continuously and is immediately reported if predefined limits are exceeded. Non-destructive techniques are usually preferred since they limit the impact on already weakened structures.

Distinction can be made according to the information content ascribed to a sensor (0D/1D/2D/3D), the number of sensors required in a network to detect and track local or global changes, but also regarding the measuring principle (electrical, (fiber-)optical, acoustic).

Acoustic Ultrasonic (US) measurement methods are characterized by the fact that a wide volume can be investigated already with a single transmitter-receiver pair. Traditional transmission and reflection methods mainly analyze the direct wave between the transmitter and receiver by means of the time of flight [7,8]. Thus, they provide information on changes or larger defects on the direct path only. Other methods focus on the late part of the US signal that follows the direct wave, the coda. The coda is scattered several times and thus interacts with larger regions. It is known to be sensitive to small changes in mechanical strain (compressive and tensile) [9,10,11,12,13,14,15], temperature [16,17,18], moisture [19,20,21] as well as cracks or other discontinuities in the concrete [22,23,24,25,26]. Since strain is the fundamental state variable of RC [27]; it seems most promising to establish a direct correlation between it and the coda to infer the health of structures.

Much effort has been spent on the material level on small compressive strains of concrete in the elastic domain [9,10,11,12,13,14]. Using the acousto-elastic effect, good agreement with US results has been found on micro-scale. By contrast, due to concrete’s significantly lower capacity in tension, purely tensile strains have seldom been investigated [15]. While the non-cracked domain ends around 30–40% of the maximum strength in compression (about 350 μstrain), in tension, it already ends at about 100 μstrain. However, from a structural point of view, both is of minor interest on the macro-scale.

Basically, RC structures are that successful due to the symbiosis of the two materials, steel and concrete. They are usually subjected to bending, which causes local compressive and tensile zones, and designed in such a way that (tensile) failure is always announced in good time and in a ductile manner. Once concrete cracks in tension, the embedded reinforcement takes all tensile forces (by bond) and enables exploiting RC’s capacity far beyond the elastic range. Macro-cracks (of limited width for durability reasons) are thus an elementary part of the construction method and by no means an exclusive indicator of imminent failure. They are inevitable, and thus, proper monitoring must cope them and only alert authorities when critical limits are reached.

The load-bearing behavior of RC after the first crack is crucial. However, previous research could only establish correlations for the non-cracked state. This article is dedicated to the (almost) entire load-bearing behavior of RC, including the cracked state.

Attempts to establish a direct correlation between strain and coda characteristics experimentally beyond the elastic range have failed so far [28]. Employed as an integral coda characteristic, the relative velocity change, obtained from coda wave interferometry (CWI) with step-wise reference, has always shown the same sign independent of stress. Thus, without augmented information employing other techniques for reference, a differentiation between compression and tension remains impossible. Nevertheless, in [28], the course of relative velocity change was found bi-linear and prominent kink at a level of about 1.3 times the cracking force. This indicates a fundamental similarity to the integral load-bearing behavior of RC structures as reflected by the well-known moment-curvature relationship [29,30,31,32].

This similarity shall be used here specifically to establish integral correlation on structural level. Abandoning everywhere highly resolved local strains, this is done in reflection of an integral but representative velocity change of a scanned partial volume between sensor pairs analogous to the specification of an average steel strain. For this purpose, an RC beam subjected to 4-point bending has been tested at Ruhr University Bochum. In addition to US measurements, the strain in the reinforcement is measured with FOS. The results of this experiment are presented here. Focus is laid on the investigation of the load-bearing behavior of RC members. An approach is presented that allows evaluating the almost entire load-bearing behavior of RC members (i.e., up to the cracked range at about 90% of the maximum load).

The article first introduces the methodology used for US measurements and discusses the key parameter computed thereof. This is followed by a mathematical description of the load-bearing behavior of RC components, and the experiments carried out. Based on these fundamentals, the load-bearing behavior is first analyzed phenomenologically (qualitatively) using various transmitter-receiver combinations. From the similarity to the material behavior of RC, a correlation relationship is developed that enables infering the member condition via US measurements.

## 2. Methods

### 2.1. Sensing Structures with Ultrasound

#### 2.1.1. General Aspects

Elastic-wave-based techniques have become an essential part of the non-destructive testing (NDT) portfolio in civil engineering. Today, methods such as the impact echo [33,34], US transmission and reflection [7,8] are well-established for material testing [35,36,37]. Measurements with US waves—waves featuring frequencies ≥20kHz—are able to analyze the thickness of components and detect major faults. Their application always requires a trade-off between resolution and penetration depth. With higher frequencies, smaller features will be detected; meanwhile, the attenuation of US is increased due to interactions with grains and pores in concrete. Planes and Larose [12] distinguish four domains for US in concrete. Differentiation takes into account the macroscopic size of the specimen *L*, the US wavelength λ, the concrete grain size *d* and the intrinsic absorption length $l_{a}$. The wavelength is a frequency *f* and velocity *v* dependent quantity defined by λ=v/f. Their four domains are limited as follows:

1 The *low frequency range* and *stationary wave regime*, where the wavelength is greater than the specimen itself. This regime is typically limited to f≤20kHz.
2 The *single scattering regime* where the wavelength is longer than the grains but smaller than the macroscopic size of the structure (d<λ<L). In this regime and 20kHz≤f≤150kHz, intrinsic absorption can be neglected since $l_{a}>L$.
3 The *multiple scattering regime* (f≤1MHz where λ<d but intrinsic absorption does not dominate signal spreading).
4 The *attenuative regime* with f>1MHz where scattering and intrinsic absorption prevent elastic waves from significant spreading.

**Figure 1:**
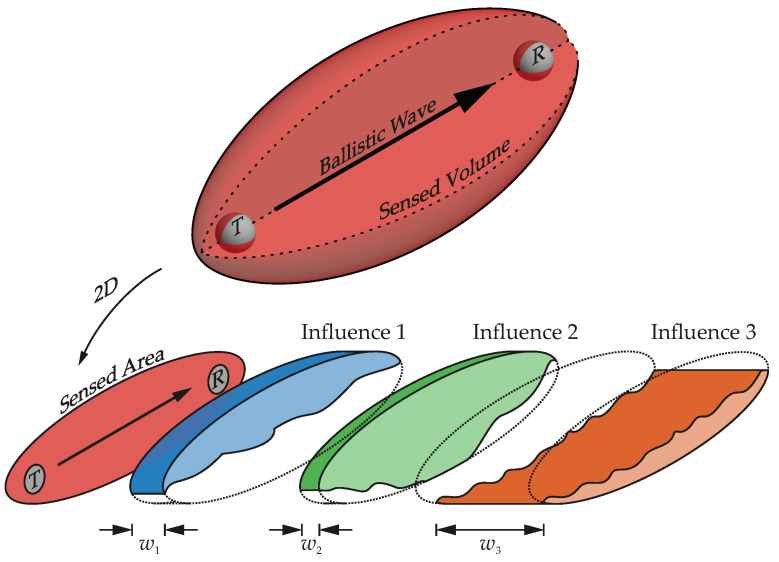
(**Top**): Simplified 3D representation of the volume scanned by US. (**bottom**): Model representation of different influences on the signal. The contribution of these influences to CWI characteristics depends on the affected region and a weighting factor.

Classical transmission-based US NDT techniques analyze ballistic waves—waves that have directly traveled from transmitter (T) to receiver (R) (see Figure 1). These techniques are situated on the lower end of the frequency ranges since they transmit significant parts of energy on the direct path. When frequency and scatter in inhomogeneous materials such as concrete increase, more wave energy is scattered and no longer travels on the direct path. Then significant portions of energy are recorded at the receiver after the ballistic part. This multiply scattered part of recorded waves is called coda [38].

Researchers in seismology and US have successfully used the coda to detect minute changes in the earth’s crust and scattering media such as concrete (e.g., [39,40,41]). Multiply scattered coda waves sense a certain volume between transmitter and receiver (Figure 1 top). Because they integrate information from the entire volume, the scattering medium acts like an interferometer that merges 3D information to a receiver location. Therefore, the coda probably joins information from different material zones with specific properties and superimposes impacts from, e.g., temperature, moisture, mechanical stress variations and permanent changes such as cracks. All influence the coda to a different degree, dependent on the geometry, the environmental conditions, and the severity of changes (Figure 1 bottom). The analysis of such superimposed information stored in the coda is called coda wave interferometry.

#### 2.1.2. The CWI-Method

**Figure 2:**
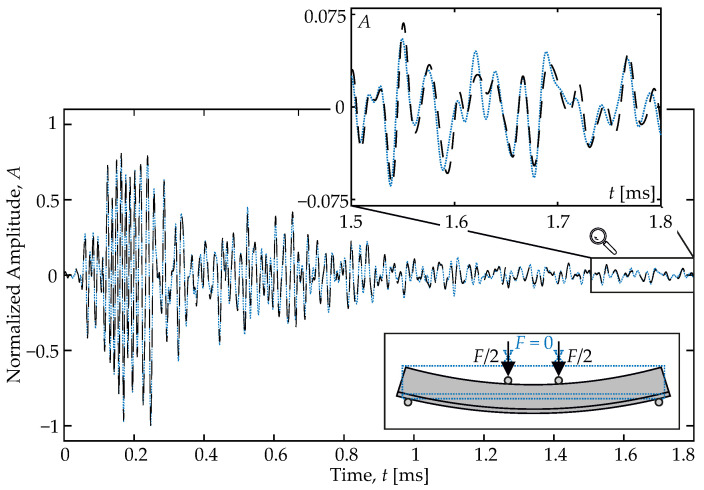
US measurement of the same transmitter-receiver combination, before and after an influence has changed.

Small changes in the coda between two consecutive measurements are analyzed by the cross-correlation of the associated signals. Figure 2 shows two consecutive measurements, where the influence of a minute change in the material parameters is only visible in the coda, but not in the first arrival. Several techniques are available to calculate characteristic velocity changes from the cross-correlation and to analyze the evolution of velocity changes in a scattering medium such as concrete (see [12]). In recent years, most researchers applied the so-called stretching technique to obtain the velocity change (e.g., [42,43,44,45]):

(1) $$-\frac{dv}{v}=\underset{\alpha \in R}{arg max}CC\left(t,\;\alpha \right)=\underset{\alpha \in R}{arg max}\frac{\int_{t_{1}}^{t_{2}}u_{1}\left(t\left(1-\alpha \right)\right)\;u_{2}\left(t\right)\;dt}{\sqrt{\int_{t_{1}}^{t_{2}}u_{1}^{2}\left(t\left(1-\alpha \right)\right)\;dt\int_{t_{1}}^{t_{2}}u_{2}^{2}\left(t\right)\;dt}}$$

As in other techniques, such as the doublet technique [12], this method compares two consecutively recorded signals $u_{1}$ and $u_{2}$. The level of similarity of these signals in a time window $\left[t_{1},\;t_{2}\right]$ is expressed by the correlation coefficient (CC); −1≤CC≤1. CC close to one indicates very similar signals. If the two signals are $180{\;}^{\circ }$ phase-shifted, CC is close to negative one. If the two signals are not similar anymore, CC approaches zero. To determine a velocity change with Equation (1) the signal $u_{2}$ is compared to a stretched (or compressed) version of $u_{1}$. The stretching factor α that maximizes CC is the relative velocity change dv/v for the sensed volume. In monitoring experiments, where several consecutive measurements are evaluated, either the initial state can be chosen as a fixed reference or the reference is updated step-wise. The latter is advantageous in cases where strong changes are expected, as strong differences between $u_{1}$ and $u_{2}$ (meaning CC<0.7) are avoided, while the relative velocity change can still be related to the initial state. Therefore, this step-wise procedure is used here throughout.

For analysis, the relative velocity change dv/v has another, yet decisive advantage over the correlation coefficient CC, since it develops strictly monotonically with time or with load. Its evolution can therefore be expected to run affine to that of common structural quantities such as strains.

As mentioned, the US coda is sensitive to small changes in the sensed material (e.g., [9,10,12,17]). When monitoring civil engineering structures, the analysis of stress (strain) changes in a structure is of special interest, as it can be an early indicator of damage. This dependency of US velocity to stress (strain) is covered in the acousto-elastic theory by the inclusion of non-linear elastic laws to the equations of motion. Then the relative velocity change depends on non-linear parameters. A detailed description of the acousto-elastic theory can be found in [46,47].

### 2.2. Load-Bearing Behavior of Flexural RC Members

Due to its low tensile strength that amounts to about 10% of its compressive strength ($f_{ct}\approx 10\%\;f_{ck}$), plain concrete is commonly not used to build structures. Its combination with (reinforcing) steel, which, in contrast, possesses significant tensile strength, compensates for this drawback. Embedded reinforcing steel is designed to take over the stresses released by concrete if necessary. While pouring, it is bonded to the concrete and usually placed in the tensile zone of flexural members (stretched fiber) expected from loading. In structural design, flexural members are commonly denoted as beams, a term that will be used synonymously in the following.

Due to global availability of raw materials, ease of workability, almost arbitrary formability, and costs, RC has become the most frequently used construction material worldwide.

#### 2.2.1. Phenomenological Characterization of RC

The composite RC composed of concrete and steel possesses a complex load-bearing behavior. As far as the top hierarchy is concerned, it can be classified into a non-cracked and a cracked domain (state I / II, cf. Equations (3)–(6)). With increasing load, RC beams pass through these states. Depending on the static system (location of the supports and the external loads) and the point of interest observed along the beam, the internal forces (stresses) also vary. As a result, the beam is usually in different states along its length (*x*), dependent on the internal stresses.

**Figure 3:**
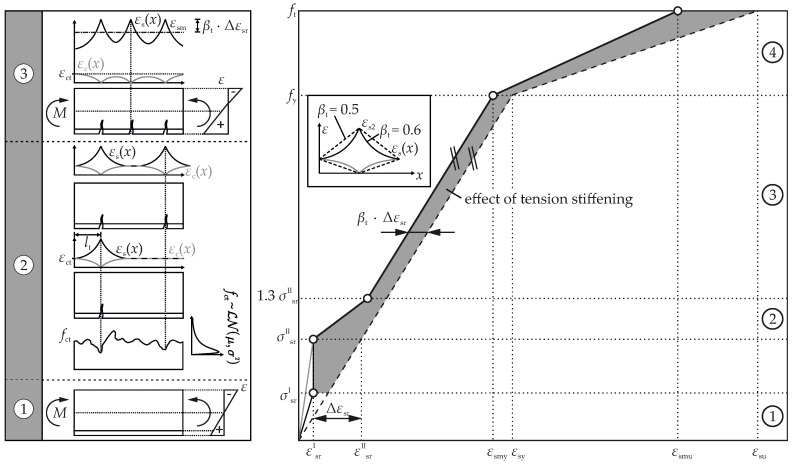
Concrete and steel strains (**left**) for the three ranges of the $\sigma_{s}$-$\varepsilon_{sm}$-relation (**right**).

As the tensile is considerably lower than the compressive strength, the load-bearing behavior is typically classified using the tensile capacity. The non-cracked state ➀ is characterized by linear elastic behavior. Both concrete and steel are entirely intact and exhibit identical strains on the same level (fiber) in the cross-section (see Figure 3 left). Then, bond stresses between the reinforcement and the concrete do not exist.

However, as the load increases, the stresses exceed the tensile strength on a certain level depending on the concrete grade, the concrete cracks. The bending crack starts from the outermost tensile fiber and runs perpendicular to the neutral axis at center.

Only cracked, RC develops its full potential. Its complex load-bearing behavior is usually further subdivided into three complementary ranges (➁ – ➃, according to e.g., [31,32,48]). In the range of cracking (➁) and since the tensile strength is subject to inherent variation, the first crack does not inevitably form at the point of maximum load (cf. Figure 3 left). Rather, the location of the first crack is always a trade-off between load and tensile strength, and thus depends to some extent on chance.

At cracks, the concrete stress and hence the strain immediately drops to 0. Then, the stress that has been borne by the concrete before is redistributed to the reinforcing steel by the bond. As a result, the stress (strain) in the reinforcement is maximum at the crack. Thus, reinforcement and concrete do no longer exhibit identical strains at the same depth (fiber). Over the transfer length $l_{t}$, the bond reintroduces the steel forces into the concrete. Finally, both strains converge on the same level again, while the rest of the beam at a distance $l_{t}$ from the crack remains non-cracked and still responds linear elastic.

When the reinforcement has transferred the entire force released by the first crack into the concrete again, the next crack emerges at the then weakest location (Figure 3 left cf. [49]). Successive cracking repeats until the stress no longer exceeds concrete’s tensile strength. This may be caused by: (1) Either the external load is no longer sufficient to exceed the tensile strength or (2) the distance between the cracks becomes too small to transfer enough force into the concrete via bond. The range of cracking (➁) ends at approximately another 30% of the force that has been necessary to generate the first crack ($1.3\cdot F_{cr}$) cf. [32].

In the third range (➂ completed crack pattern), no new cracks form anymore. The crack spacing is final and lies between $l_{t}$ and $2\;l_{t}$. With increasing load, the existent cracks widen while the steel strain rises. At some point (➃), the yield strength of the reinforcing steel is exceeded in a crack (location is again a trade-off between yield strength and load), the reinforcement yields. No more stresses or forces can be overtaken. From now, load increase goes along with significant elongation and strains and thus crack width. Neglecting potential failure of concrete in the compressive zone (stresses exceed the maximum compressive strength), the beam fails when reaching the tensile strength of the reinforcement at some location. This failure is strongly ductile since it is well-announced by significantly increasing strains before.

#### 2.2.2. Intermediate Resume

*Crack before failure* is the fundamental design principle of RC. Thus, RC structures are always designed on the safe side, assuming the purely cracked state and neglecting any contribution of concrete to the tensile capacity of the member. In the check of equilibrium of internal forces, all stresses in the compressive zone are ascribed to the concrete while tensile stresses are appointed to the reinforcement. However, what is safe and simple in the design is too extensively idealized regarding realistic deflection prognoses on service level. Taking that simplified assumption, the actual deflection would be strongly overestimated.

Thus, in service conditions, it is of utmost importance to carefully take into account the concrete’s contribution to the tensile capacity. That capacity of concrete between the cracks, which increases the total stiffness of the member, is often referred to as tension stiffening. Two alternatives are established to take it into account. One distinguishes a concrete-based formulation [29,30] from a steel oriented approach [31,32,48,50]. The latter formulation via an average steel strain (cf. Figure 3 right) is used here since both the steel strain and relative velocity change, are integral properties of associated characteristic volumes and alter during cracking. This correlation shall be exploited.

#### 2.2.3. Computational Approach

Mathematically, the—due to cracking—fluctuating steel strain along the beam $\varepsilon_{s}\left(x\right)$ can be averaged. The average steel strain $\varepsilon_{sm}$ is obtained from Equation (2) by integration and division through the integration length. That way, realistic steel strains for serviceability conditions can be computed.

(2) $$\varepsilon_{sm}=\frac{1}{l_{t}}\int_{0}^{l_{t}}\varepsilon_{s}\left(x\right)\;dx$$

Please recall that the calculation of strains in the non-cracked state ➀ is elementary. Without cracks, the strain along the beam $\varepsilon_{s}^{I}$ varies only according to the external forces. On each location, its distribution over the depth is linear and zero at the neutral axis. Upwards, this range is limited by the steel stress associated with concrete cracking $\sigma_{sr}^{I}$.

(3) $$➀:\;\varepsilon_{sm}=\varepsilon_{s}^{I}\;\mathrm{for}\;0<\sigma_{s}\leq \sigma_{sr}^{I}$$

For convenience, the following range of cracking ➁ is first skipped. First, focus is set on the range of completed cracking ➂. When cracked, the force between concrete and steel is transferred by bond. Bond is mathematically captured by a bonding law that generally idealizes the location and slip-dependent shear force transfer between concrete and rebar. It describes how the steel strain in the vicinity of a crack develops. The average steel strain in range ➂ follows from Equation (4).

(4) $$➂:\;\varepsilon_{sm}=\varepsilon_{s}^{II}-\beta_{t}\cdot \Delta \varepsilon_{sr}\;\mathrm{for}\;1.3\cdot \sigma_{sr}^{II}<\sigma_{s}\leq f_{y}$$

Herein, $\beta_{t}$ governs the postulated bonding behavior, i.e., it indicates the steel stress development starting from the crack. Taking the simplest assumption $\beta_{t}$ is constant, the bonding behavior is constant, too. This explicitly means: it does not change with load increase. More accurate approaches to account for changing bond properties due to local cracking are published elsewhere [32,48,51]. The strain gain in the reinforcement at transition from the non-cracked to the cracked state is denoted $\Delta \varepsilon_{sr}$.

Thus, in view of Equation (4) and Figure 3 right, it is inferred that the term $\beta_{t}\cdot \Delta \varepsilon_{sr}$ corrects the purely cracked state for tension stiffening. Moreover, in terms of stress, region ➂ is bounded to the top by yielding of the reinforcement ($\leq f_{y}$). Towards the bottom, it is limited by transition to the range of cracking ($>1.3\cdot \sigma_{sr}^{II}$). From compatibility to the ranges ➀ and ➂, the average steel strain in the range of cracking ➁ is deduced which yields Equation (5).

(5) $$➁:\;\varepsilon_{sm}=\varepsilon_{s}-\frac{\beta_{t}\cdot \left(\sigma_{s}-\sigma_{sr}^{II}\right)+\left(1.3\cdot \sigma_{sr}^{II}-\sigma_{s}\right)}{0.3\cdot \sigma_{sr}^{II}}\cdot \Delta \varepsilon_{sr}\;\mathrm{for}\;\sigma_{sr}^{II}<\sigma_{s}\leq 1.3\cdot \sigma_{sr}^{II}$$

When yielding has occurred (index *y*), the ultimate load-bearing capacity of the beam associated with $f_{t}$ is almost reached. With just small gains in the stress level ($f_{t}/f_{y}\approx$ 105–110%), strains and deformation in range ➃ grow fast. Then, the average steel strain is obtained from Equation (6) wherein $\delta_{d}$ accounts for the ductility of the reinforcement ($\delta_{d}=0.8$ high ductility and $\delta_{d}=0.6$ normal ductility).

(6) $$➃:\;\varepsilon_{sm}=\varepsilon_{sy}-\beta_{t}\cdot \Delta \varepsilon_{sr}+\delta_{d}\cdot \left(1-\frac{\sigma_{sr}^{II}}{f_{y}}\right)\cdot \left(\varepsilon_{s}-\varepsilon_{sy}\right)\;\mathrm{for}\;f_{y}<\sigma_{s}\leq f_{t}$$

Considering all four ranges, a piece-wise defined function of the average steel strain is available that covers all phenomenological aspects introduced above. Elsewhere, it has already been applied to compute realistic deformations of a wide variety of RC structures [52,53,54]. For this purpose, the average steel strain must first be converted into an average curvature and then (numerically) integrated. Through additional deliberations, the same approach has also been extended to cover other, even more difficult, composite materials such as steel fiber reinforced concrete SFRC [55,56].

## 3. Experiments

### 3.1. Experimental Setup

**Figure 4:**
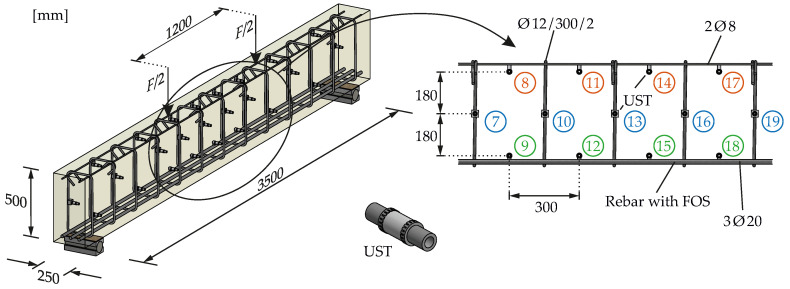
Beam geometry, reinforcement layout and loading along with the US transducer (UST) network.

To substantiate the supposed principle correlation between the average steel strain and the relative velocity change as a key property of ultrasound obtained from CWI, an experiment has been set up at the lab at Ruhr University Bochum. One simply supported RC beam with rectangular cross-section shown in Figure 4 and dimensions 250/500/3500 [mm] (width/depth/field length) subjected to four-point bending was manufactured. To cover bending and shear demands with respect to EC 2 [57], it has been equipped with longitudinal reinforcement (3Ø20 mm) and stirrups (Ø12 mm/300 mm/2). This simple setup meets the experimental objectives quite well since the central shear-free zone between the two vertical loads yields a plateau of constant bending moment. Herein, pure bending induces constant strains in each fiber and a neutral axis at the center. The strain distribution over the depth is linear. Thus, the principal stresses are strictly orientated horizontally along the beam. Interference with shear (indicated by inclined principal stresses) must not be expected. In this region, a distributed crack pattern with random characteristics as discussed above (cf. Section 2.2.1) will develop while the external load remains simple and controllable. During the test, the load will be increased step-wise and slowly to track all intermediate changes and to record the strains up to bending failure.

### 3.2. Placement of Measuring Equipment

**Figure 5:**
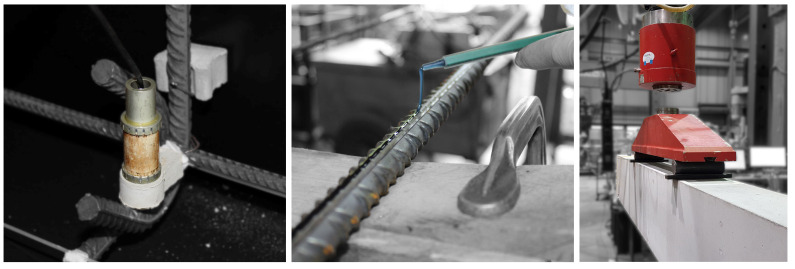
(**Left**): US transducer attached to the reinforcement with a quick-cement fastener. (**center**): Gluing a FOS to the rebar. (**right**): Beam before the experiment in the test bench.

To record US data, a transducer network has been installed all along the beam (Figure 4). It covers the central region of the beam on three levels. At the bottom and top layers, the transducers are attached to the longitudinal and constructive reinforcement (2Ø8 mm, cf. Figure 4), respectively. The central layer is fixed to the stirrups. Embedded US transducers (type SO807 from Acoustic Control Systems (Sarrebruck, Germany), Ltd.) were used. They consist of a piezoceramic cylinder and have a central frequency of approximately 60 kHz. The radiation was already characterized in [58,59] and is approximately uniform in the plane perpendicular to the piezo ring. Newly developed fasteners made from quick-cement (see Figure 5 left) fix the transducers to the reinforcement. Since their material is similar to the beam’s concrete, they do not significantly impair wave propagation and load-bearing behavior.

For reference, a broad spectrum of strain measurement techniques (strain gauges, digital image correlation DIC, FOS, etc.) is available [60]. However, not all methods are equally suited. Conventional strain gauges face difficulties in allocating the measured data to a distinct position. Due to the physical length of a gauge about 3 to 6 mm, the strain is always averaged over that length. So the resolution is limited by the length. Another limiting factor concerns the number and density of individual gauges glued on concrete or steel surfaces and the associated effort. Furthermore, gauges always deliver highly local one-dimensional directed information. Moreover, since the decision where a gauge is to be placed has to be taken in advance (long before the experiment starts), the chance is great to miss the strain hot-spot at a crack whose location is random by nature as discussed above (cf. Section 2.2.1). These deficiencies leave strain gauges marginally relevant for the intended application. However, they yield highly precise local strain references to double-check or calibrate other devices.

Thus, measurement techniques that provide higher information density (1D to 3D strain data) seem to be essential. With DIC, exclusively two-dimensional surface strains are observable, which is good for concrete strains but makes the acquisition of steel strains of embedded reinforcement infeasible. Furthermore, strain measurement with FOS is a trade-off. One-dimensional strains along optical fibers are obtained in (sub)millimeter resolution. When appropriately applied (gluing in a groove in the rebar [28,60,61], see Figure 5 center), strains can be acquired along entire reinforcement bars in high resolution. Installation effort and precision depend on the right combination of core, coating and cladding [60].

### 3.3. Concreting and Curing

Approx. 0.8 m^3^ of concrete was mixed for the test specimen and several concrete cubes and cylinders (to determine the material properties). The fresh concrete was poured into the prepared formwork and compacted with the aid of a vibrating table. After 28 days of curing, the test was carried out. On the test day itself, the accompanying material tests (concrete and reinforcement) gave the material parameters listed in Table 1. All values are an average of three individual samples tested.

### 3.4. Load Control

Moreover, strain measurements with FOS can be performed quasi continuously (at defined intervals). In contrast, US measurements require load steps during testing since they measure differences between load stages. On each load level (under constant load), the US measurements are conducted transmitting from one US transducer and receiving at another. Successively all other combinations are worked through before the load is increased to the next level.

As discussed above, the time of cracking (end of the range ➀, corresponding load $F_{cr}$) is a tipping point for both the load-bearing behavior of RC and the results of ultrasound. For this reason, the load is finer graded in the initial range. Up to $2\cdot F_{cr}\approx 100$ kN, it is increased in 5 kN steps. Thereafter, the increment is doubled until the reinforcement yields (end of range ➂, corresponding load $F_{y}\approx 340$ kN). The significantly reduced stiffness of the specimen beyond this level rapidly leads to inconstant strains when yielding even on a fixed load level. Then US results can no longer be assigned to a distinct load level without doubt, and testing is consequently stopped.

The environmental conditions in the laboratory are held constant at approx. 22 °C and 52% relative humidity.

### 3.5. Proof of Concept

**Figure 6:**
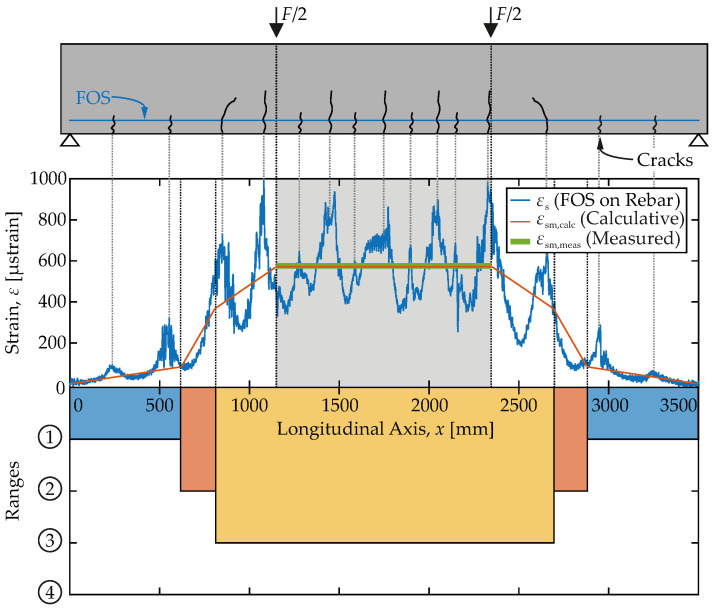
(**Top**): Strain records of the FOS on the flexural reinforcement compared to theoretically expected average steel strains. (**Bottom**): Associated ranges employing the $\sigma_{s}$-$\varepsilon_{sm}$-relation on an equivalent load level.

With the FOS on the flexural reinforcement, strains are quasi continuously sensed along the entire beam (spacing of measurement points <1 mm). Figure 6 displays such strain readings in the reinforcement (in blue) for a load of F=100 kN. Cracks in the concrete are clearly indicated by the peaks. They are induced by the strains transferred from the reinforcement through bond. Their spacing is regular, as can be expected for the range of completed cracking ➂. From the strain record, it can be seen that at that load, cracks have occurred almost over the entire specimen, not only in the central region of constant moment between the loads. Besides a rapid increase of the strains around the cracks, the curve is characterized by scatter. However, the magnitude of the scatter is small compared to the strain peaks.

For comparison the average steel strain in the reinforcement (orange) is computed employing Equations (3)–(6). Throughout the piece-wise defined mean function idealizes the measured course quite well. Depending on the bending moment in longitudinal direction, individual parts of the beam are assigned to the different ranges. The non-cracked region close to the supports is followed by the range of cracking ➁ and the cracked region with completed crack-pattern ➂ at center.

In the measuring field between the loads, the strain is constant due to the constant bending moment. Here, the average steel strain (green) can be obtained, analog to Equation (2), from averaging the FOS readings over the measuring length, too.

(7) $$\varepsilon_{sm}=\frac{1}{1200\;\mathrm{mm}}\int_{1150\;\mathrm{mm}}^{2350\;\mathrm{mm}}\varepsilon_{s}\left(x\right)\;dx$$

This average strain ($\varepsilon_{sm,meas}=571$ μstrain) is plotted in green in Figure 6. It nearly coincides with the theoretically predicted mean from calculation ($\varepsilon_{sm,calc}=572$ μstrain, orange). No doubt, at other load levels, the difference may be greater. However, in general computational prediction of material behavior by Equations (3)–(6), albeit idealizing, seems suitable and in good agreement with the high-resolution measurements by FOS.

## 4. Results and Discussion

### 4.1. Development of the Relative Velocity Change with Time and Load

**Figure 7:**
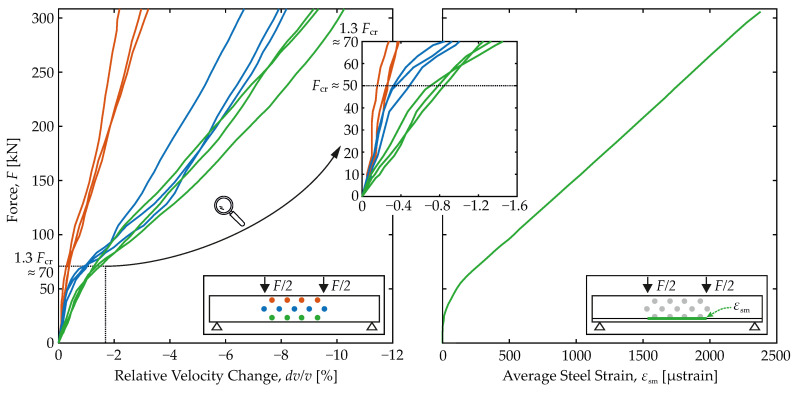
Load of the specimen plotted versus the relative velocity change (**left**) with particular focus on the non-cracked ➀ and cracking ➁ range and average steel strain (**right**).

On the left in Figure 7, the force *F* on the specimen is plotted versus the relative velocity change dv/v from CWI. All results of transmitter-receiver combinations from the compressive (orange) and tensile (green) zones, as well as the neutral axis (blue), are displayed. However, the selection has been limited to the next neighbors (sensor distance 30cm) of the horizontal combinations only. For each zone, three curves are plotted since one sensor in the neutral axis failed.

Due to the constant bending moment between the concentrated loads, all three combinations on each level form a set and theoretically represent identical states. In the non-cracked state, this is more easily recognized, since a homogeneous stress distribution theoretically exists throughout. Certainly, in the cracked state, this expectation seems justified, too, due to the uniformly distributed cracks caused by the load pattern and the integral sensing of the material by the US signal.

Consequently, no further distinction must be made between the individual curves on the same level. They all have the same features and show the same characteristics. Deviations of the curves in a set from each other are attributed to inherent scatter, while the differences between the sets are seen significant in view of material response.

In general, all curves in the diagram show two almost linear branches and a range of transition in between. In the range from 0 to 50 kN, they have an initial slope which becomes flatter in transition between 50 and 75 kN and then remains approximately constant until the end. The change in slope is most pronounced for the tensile curves and weakest for the compressive ones. The US signal is more affected in the tensile range than in the compressive range, which is attributed to concrete cracking. Regarding their limits, the three ranges agree with the known limits of the material behavior.

Great similarity to the average strain in the reinforcement computed from the FOS readings according to Section 3.5 can be observed in Figure 7 on the right. Therein, the evolution of the average steel strain with load is shown. The parallelism between the characteristics of the *F*-dv/v relation and those of the $\varepsilon_{sm}$-$\sigma_{s}$ relation becomes apparent. The following conclusions are drawn:

1 Cracking strongly affects the US signal and the key quantities computed with CWI, the correlation coefficient CC and the relative velocity change dv/v.
2 The relative velocity change is always negative.
3 Characteristic points associated with significant changes in the material response $F_{cr}$ and $1.3\cdot F_{cr}$ can be identified in the *F*-dv/v relation, too.
4 A fundamental change of the load-bearing behavior happens in the range of cracking ➁ and is also predicted by the *F*-dv/v relation.
5 In agreement with the decreasing slope of the $\varepsilon_{sm}$-$\sigma_{s}$ relation, the slope of the *F*-dv/v relation decreases in transition from the non-cracked to the cracked state.

As expected and illustrated in Figure 7, the curves of sensor pairs in the neutral axis fall between the compressive and tensile sets of curves. More precisely, they are first oriented towards the curves of the compressive set. However, then, with cracking, they tend to follow the tensile set, which leads to the following conclusions:

1 The closer a sensor pair is located to the tensile zone, the more the relative velocity change drops.
2 With load increase, cracks gradually grow towards the compressive zone at top. This is well-reflected by the trend of the central axis’ gradient towards the tensile one. As the cracks approach the centerline transducers, the US signal is affected more like the signal in the tensile zone.
3 Not even in the non-cracked state with linear elastic material behavior, the central trend is the average of compressive and tensile trends. Thus, the relative velocity change develops non-linear with the load.

Confined to purely mechanical considerations, influences from elastic strain and, above all, from cracks are inseparably linked to the signal. The scanned volume (cf. Figure 1) integrates and mixes impacts from unloaded, elastically loaded and cracked regions. Their proportions are reflected in the magnitude of the relative velocity change.

Moreover, uncertain onset of cracking, load-dependent crack growth, as well as the variable compressive and tensile zone heights, cause these proportions to vary with time and load. Different sensitivity to tensile and compressive strains in general and with respect to the material state, cracked or non-cracked, is another factor that affects the magnitude of the relative velocity change.

### 4.2. Establishing a Correlation Function

**Figure 8:**
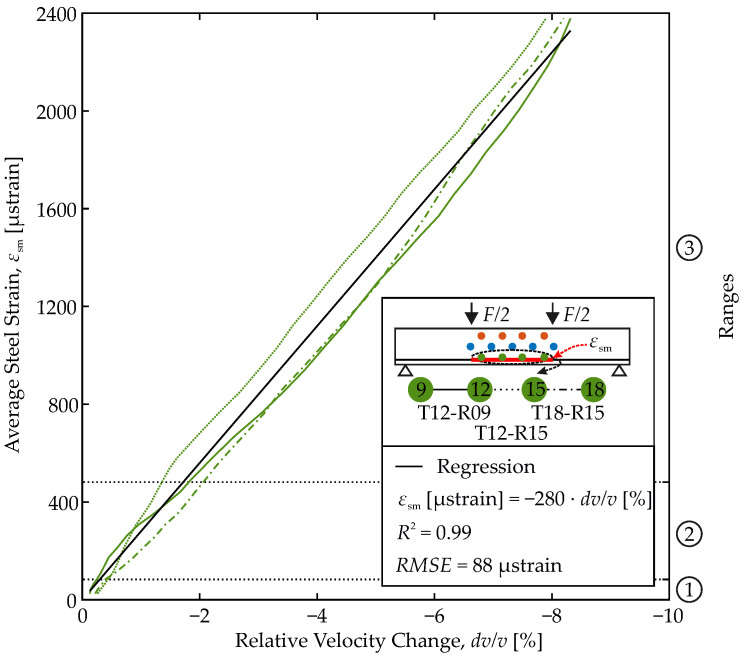
Correlation of average steel strain to relative velocity change.

Figure 8 combines the average steel strain computed from the FOS results and the relative velocity changes of the US transducer pairs in the tensile zone from Figure 6. Both are coupled via the load level. Three nearly straight curves are obtained. Even at first glance, average steel strain and relative velocity change appear to be linearly correlated. By linear regression, the correlation function according to Equation (8) can be established with great confidence.

(8) $$\begin{array}{cc} \varepsilon_{sm} & =c\cdot dv/v\\ \; & \mathrm{with}\;c=const.\;\left[\frac{\mu \mathrm{strain}}{\%}\right] \end{array}$$

Therein, strain is to be inserted in μstrain and the relative velocity change in %. The constant *c* has to be determined on each individual member. In our case *c* equals −280μstrain/%. This model yields a coefficient of determination of 0.99 explaining 99% of the strain variation by variation of the relative velocity change. The error is small and quantified to 88 μstrain on average by the Root Mean Squared Error (RMSE).

The model is ready to be used to infer from US measuring data and thereof computed relative velocity changes on the average steel strain in the sensed member.

Finally, the model’s range of applicability shall be discussed. In the experiment, the load is increased step-wise up to 300 kN at maximum. Up to this load level, relative velocity changes and reinforcement strains are carefully tracked. From the member’s design according to EC 2 [57], yielding of the reinforcement is expected at a load level of 340 kN, while the ultimate load-bearing capacity is reached at 375 kN. Thus, the model covers 80% of the permissible load range.

Since no significant changes in the load-bearing behavior are to be expected from mechanical reasons until the reinforcement yields, the model can even be used beyond the experimentally validated range. With yielding, 90% of the permissible load range would finally be covered by extrapolation. Both go significantly beyond any other correlations of ultrasonic and structural parameters of RC members found so far, which are all limited to the linear elastic range.

### 4.3. Impact of the Transducer Distance

The proposed model is based on US measurement data from transducer pairs of 30 cm distance throughout. This distance is not particularly long, neither with regard to usual RC members, nor (and even more so) with regard to the monitoring of entire structures. For reasons of costs and installation, the aim is to use fewer, i.e., more distant, sensors. In construction practice, the optimum is often a compromise between costs and density of the sensor network. Therefore, it has finally been investigated whether a tripled/quadrupled transducer distance has an impact on the obtained results or the found model.

**Figure 9:**
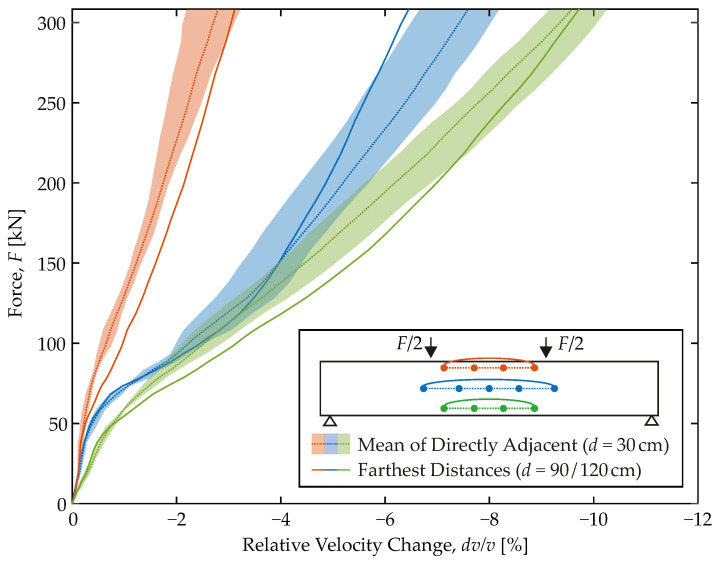
Fitting of longer transmitter-receiver combinations in the presented shorter ones.

The issue is addressed in Figure 9. At first, a mean response (dashed line) is computed from the results of the three sensor pairs per level already shown in Figure 7. Moreover, a shaded band of scatter is provided, that marks the minimum and maximum relative velocity changes on all load levels. Both rest on the short transducer distance of 30 cm.

From the recorded US data, the results for the longest transducer distances are then computed analogously. They are shown as solid lines and cover distances of 90 up to 120 cm. All three follow the mean courses and preserve the key characteristics of the initial curves. No clearly deviating trends can be identified. Thus, it is concluded that even with greater transducer distances, a similar model would be obtained. As long as this is not available, the proposed model might be used instead. One must not expect significant deviations in the proposed steel strains.

## 5. Conclusions

In the context of an ever-increasing need for structural health monitoring of existing RC structures, the paper links essential key parameters computed from the coda of US signals to structural state variables and their evolution with time and load. Based on empirical data obtained on a simply supported RC beam subjected to four-point bending, a linear model is derived that correlates the relative velocity change of the coda dv/v to the mean strain of the reinforcement $\varepsilon_{sm}$ from FOS. Mechanically, $\varepsilon_{sm}$ integrates all characteristics of RC structures as known from moment-curvature relations and enables infering the material state consistently. In detail:

- For the first time, a correlation between a key parameter of ultrasound and a structural state variable is established that covers 90% of the complex load-bearing behavior of RC members. It integrates load-dependent stiffness and concrete cracking.
- For the beam and a sensor distance of 30 cm, a linear model is established with great confidence: $\varepsilon_{sm}=c\cdot dv/v$ with: $c=-280,\;R^{2}=0.99,\;RMSE\approx 90\;\mu$strain.
- Tripled sensor distances do not impair the proposed correlation function much.
- Whether in compression or tension, dv/v obtained from step-wise CWI is found to be negative throughout.
- The closer a sensor pair is located to the tensile zone, the more dv/v drops.
- Although dv/v always integrates elastic effects as well as micro- and macro-cracking, the latter seems to dominate. Compared to the compressive zone, dv/v is more sensitive to changes in the tensile zone.
- Conversely, changes in the compressive zone are more difficult to detect because they are easily superimposed by tension.

Of course, a general, component-independent correlation relationship for arbitrary RC members has not yet been established. Just a possible approach to achieve this goal has been shown. To achieve this, further experiments are certainly needed, which have to cover not only structural quantities or different concrete composition but also environmental conditions and changes of these. The wide range of influences on the US signal has to be considered for real-world application of this approach. With the consideration of environmental conditions and structural influences, the presented approach can be used to infer the loading of the RC structure by US measurements. A statement about its health becomes possible. 

## Figures and Tables

**Table 1:** Material properties of the concrete and reinforcement, respectively.

$f_{cm}$	$f_{ctm}$	$E_{cm}$	$f_{y}$
$\left[N/{\mathrm{mm}}^{2}\right]$	$\left[N/{\mathrm{mm}}^{2}\right]$	$\left[N/{\mathrm{mm}}^{2}\right]$	$\left[N/{\mathrm{mm}}^{2}\right]$
33.0	2.8	28,800	552.0

## Abbreviations

The following abbreviations are used in this manuscript:

FOS	Fiber Optic Sensor
US	Ultrasonic
RC	Reinforced Concrete
NDT	Non-Destructive Testing
CWI	Coda Wave Interferometry
